# Mesenchymal stem cell-derived apoptotic bodies alleviate alveolar bone destruction by regulating osteoclast differentiation and function

**DOI:** 10.1038/s41368-023-00255-y

**Published:** 2023-12-01

**Authors:** Xiaoyan Li, Yiyang Jiang, Xu Liu, Jingfei Fu, Juan Du, Zhenhua Luo, Junji Xu, Ujjal Kumar Bhawal, Yi Liu, Lijia Guo

**Affiliations:** 1 Laboratory of Tissue Regeneration and Immunology and Department of Periodontics, Beijing Key Laboratory of Tooth Regeneration and Function Reconstruction, School of Stomatology, Capital Medical University, Beijing, China; 2 Research Institute of Oral Science, Nihon University School of Dentistry at Matsudo, Chiba, Japan; 3 Center for Global Health Research, Saveetha Medical College and Hospitals, Saveetha Institute of Medical and Technical Sciences, Saveetha University, Chennai, Tamil Nadu India; 4 Department of Orthodontics School of Stomatology, Capital Medical University, Beijing, China

**Keywords:** Mesenchymal stem cells, Periodontitis, Cell biology

## Abstract

Periodontitis is caused by overactive osteoclast activity that results in the loss of periodontal supporting tissue and mesenchymal stem cells (MSCs) are essential for periodontal regeneration. However, the hypoxic periodontal microenvironment during periodontitis induces the apoptosis of MSCs. Apoptotic bodies (ABs) are the major product of apoptotic cells and have been attracting increased attention as potential mediators for periodontitis treatment, thus we investigated the effects of ABs derived from MSCs on periodontitis. MSCs were derived from bone marrows of mice and were cultured under hypoxic conditions for 72 h, after which ABs were isolated from the culture supernatant using a multi-filtration system. The results demonstrate that ABs derived from MSCs inhibited osteoclast differentiation and alveolar bone resorption. miRNA array analysis showed that miR-223-3p is highly enriched in those ABs and is critical for their therapeutic effects. Targetscan and luciferase activity results confirmed that Itgb1 is targeted by miR-223-3p, which interferes with the function of osteoclasts. Additionally, DC-STAMP is a key regulator that mediates membrane infusion. ABs and pre-osteoclasts expressed high levels of DC-STAMP on their membranes, which mediates the engulfment of ABs by pre-osteoclasts. ABs with knock-down of DC-STAMP failed to be engulfed by pre-osteoclasts. Collectively, MSC-derived ABs are targeted to be engulfed by pre-osteoclasts *via* DC-STAMP, which rescued alveolar bone loss by transferring miR-223-3p to osteoclasts, which in turn led to the attenuation of their differentiation and bone resorption. These results suggest that MSC-derived ABs are promising therapeutic agents for the treatment of periodontitis.

## Introduction

Periodontitis is a common disease characterized by the destruction of supporting alveolar bone, followed by an inflammatory host response, secondary to infections by periodontal bacteria.^1–3^ The pathogenesis of periodontitis involves the imbalance between bone formation and resorption. Osteoclasts differentiate from monocytes/macrophages and macrophage colony stimulating factor (M-CSF) and RANKL produced by osteoblasts mediate the generation of osteoclasts. Osteoclast precursor cells express RANK (RANKL receptor) and recognize RANKL expressed by osteoblasts through cell-cell interactions and differentiate into osteoclasts in the presence of M-CSF.^4–6^ Over-activated osteoclasts are the key factor involved in periodontal destruction.

Mesenchymal stem cells (MSCs) residing in periodontal tissues are critical for periodontal regeneration. MSCs are undifferentiated cells with capabilities for self‐proliferation and multi‐lineage differentiation that have a promising impact for the treatment of bone destruction.^7–9^ Several studies have demonstrated that MSCs rescue bone destruction by homing to the damaged areas, then differentiating into osteoblasts and/or functioning in a paracrine manner.^10,11^ The regenerative treatment of periodontitis is based on controlled inflammation. However, due to the colonization of Gram-negative anaerobic pathogens, damage of the surrounding vasculature and respiratory bursts of immune cells in the periodontal tissue, a lower‐oxygen microenvironment is a common feature of periodontitis.^12–15^ Hypoxia has been shown to induce the apoptosis of MSCs.

Apoptosis is one type of programmed cell death. During apoptosis, apoptotic cells generate various types of extracellular vesicles, including exosomes, microvesicles and apoptotic bodies (ABs), the latter being the major product of apoptotic cells. ABs range from 1 to 5 μm in diameter and can be engulfed by macrophages, dendritic cells, epithelial cells, endothelial cells and fibroblasts.^16–20^ ABs contain cytosolic proteins, lipids and genetic factors, such as mRNAs, miRNAs and lncRNAs,^21–25^ which are involved in immune activation, the recruitment of apoptotic cells and in tissue regeneration. After ABs are engulfed by their target cells, their contents are released and then regulate downstream receptor cells for intercellular communications. Emerging studies have shown that ABs can be used as promising tools for various treatments, such as *Staphylococcus aureus* infections, atherosclerosis, hepatic fibrosis, diabetes, wound healing, etc.^26–32^ However, whether MSC-derived ABs affect osteoclasts and/or periodontitis was unknown.

In this study, the effects of ABs secreted from MSCs on periodontitis in an animal model were investigated. The results showed that ABs significantly contributed to the suppression of osteoclast differentiation and the progression of osteoclastogenesis via the transfer of miRNAs. The mediator for targeted phagocytosis of ABs by osteoclasts was identified. Collectively, the results of this study show that MSC-derived ABs are a potential promising therapeutic agent for the treatment of periodontitis.

## Results

### Bone marrow MSCs undergo apoptosis in the periodontal hypoxic microenvironment

**Fig. 1:**
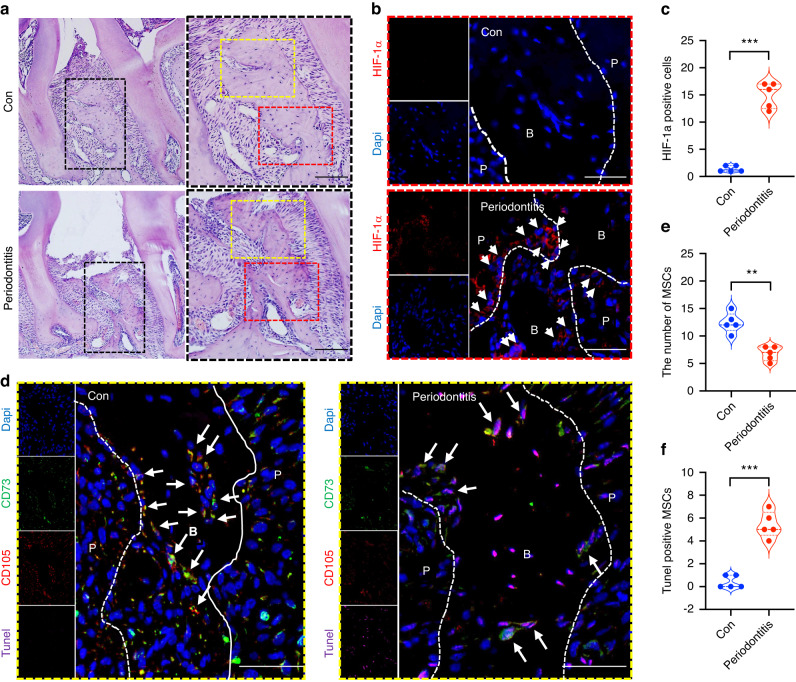
MSCs derived from mouse bone marrow undergo apoptosis in the periodontal hypoxic microenvironment. **a**–**c** The expression level of HIF-1a is significantly induced in the periodontitis group compared with the control group, which indicates the hypoxic microenvironment in periodontal tissue. **d**–**f** Under hypoxic conditions, the number of MSCs (CD73 and CD105 positive cells) in alveolar bone is reduced in the periodontitis group (showed in **e**), and consistently, the number of Tunel-positive MSCs is increased (showed in **f**). Scale bars: 50 μm. **P < 0.01, ***P < 0.001

A periodontitis animal model was established using mice to understand the fate of MSCs during periodontitis. The expression level of HIF-1a was significantly induced in periodontal tissue of the periodontitis group compared with the control group, which indicates the hypoxic microenvironment (Fig. 1a–c). Under hypoxic conditions, the number of MSCs in the alveolar bone was reduced in the periodontitis group, and consistently, the number of Tunel-positive MSCs was increased (Fig. 1a, d–f). These results revealed that MSCs underwent apoptosis in the hypoxic microenvironment that was induced by periodontitis.

### ABs inhibit osteoclast differentiation and function

**Fig. 2:**
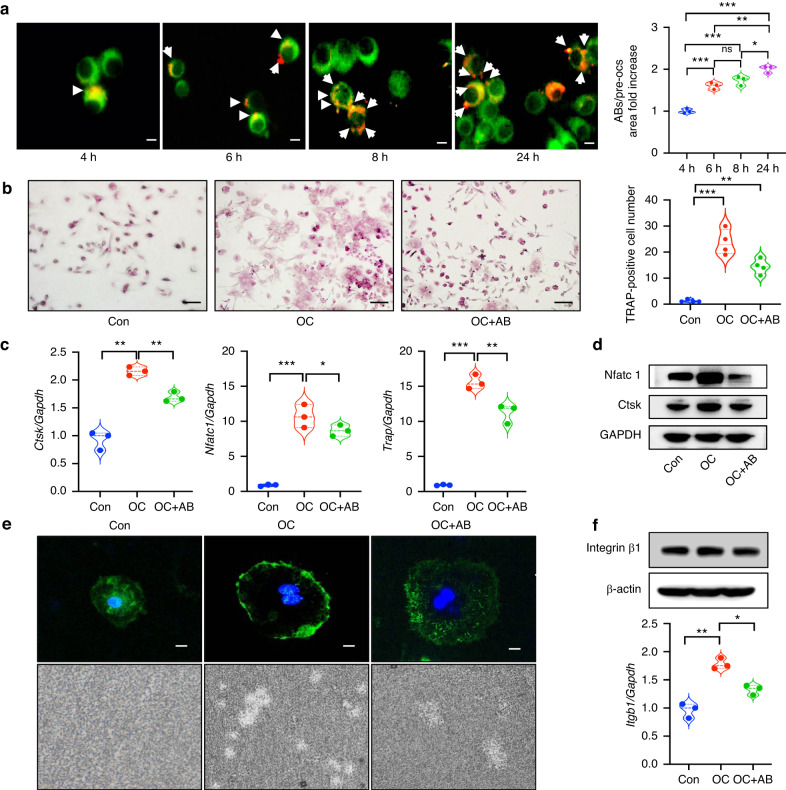
ABs inhibits osteoclast differentiation and function. **a** ABs are engulfed by pre-OCs and accumulate in those cells over time and the engulfed ABs after co-culture was quantified. **b** Compared with the osteoclast induction group, ABs significantly inhibit the differentiation of TRAP-positive osteoclasts. **c**, **d** The expression levels of Cathepsin K (Ctsk) and Nfatc1 are reduced in the AB group. **e** The sealing zone formation and bone resorption ability of osteoclasts is diminished in the presence of ABs. **f** ABs have reduced levels of integrin β1 and interfere with the osteoclastogenesis process. Scale bars: 20 μm. Data are reported as means ± SD (*n* = 3). *P < 0.05, **P < 0.01, ***P < 0.001

MSCs were cultured under hypoxia for 72 h to induce apoptosis in vitro (Figs. S1–2). ABs were isolated and identified (Fig. S3). To understand the effects of ABs on osteoclasts, ABs were co-cultured with pre-osteoclasts (pre-OCs). ABs were engulfed by pre-OCs and accumulated in those cells over time (Fig. 2a). Compared with the osteoclast induction group, treatment with ABs significantly inhibited the differentiation of OCs. The number of TRAP-positive cells decreased (Fig. 2b) and the expression levels of Cathepsin K (Ctsk) and Nfatc1 (Fig. 2c, d) was reduced in the AB-treated group. To further examine the effects of ABs on osteoclastogenesis, we evaluated bone resorption activity using Pit assays. As shown by the diminished area of resorption pits formed by osteoclasts, the bone resorption ability of osteoclasts was weakened in the presence of ABs (Fig. 2e). The expression of integrin β1 was induced during osteoclast maturation, however, treatment with ABs reduced the level of integrin β1 and interfered with the osteoclastogenesis process (Fig. 2f). ABs not only inhibited osteoclast differentiation, but also disturbed the functions of osteoclastogenesis.

### Treatment with ABs alleviates alveolar bone destruction

**Fig. 3:**
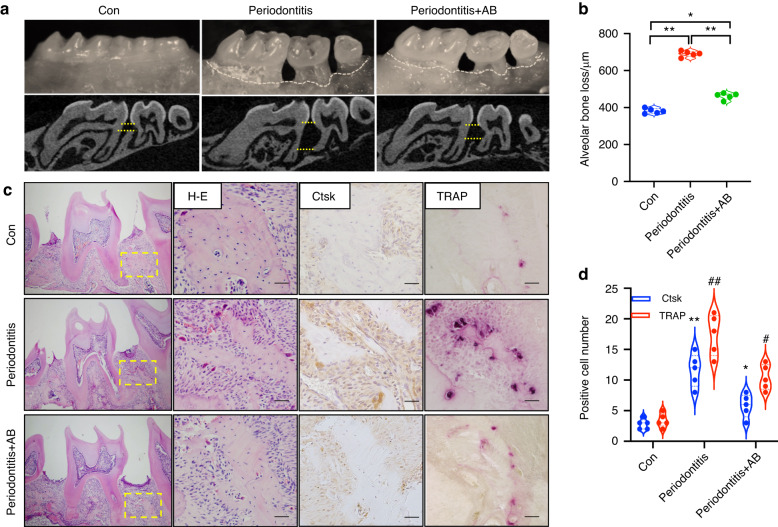
Treatment with ABs alleviates alveolar bone destruction. **a**, **b** ABs significantly alleviate the bone resorption measured by micro-CT. Upper yellow line: Cementoenamel junction; lower yellow line: alveolar crest. **c**, **d** The injection of ABs inhibits the expression level of Ctsk and the number of TRAP-positive cells as shown by immunohistochemistry and Trap staining, respectively, followed by restrained differentiation of osteoclasts. Scale bars: 20 μm. Data are reported as means ± SD (*n* = 5). *P < 0.05, **P < 0.01

In order to examine whether ABs could rescue alveolar bone destruction, we established a periodontitis model in mice. ABs were injected into the gingival sulcus and significantly alleviated the bone resorption measured by micro-CT (Fig. 3a, b). Further, the differentiation of osteoclasts was highly induced, but the injection of ABs restrained that process and the number of osteoclasts was reduced compared with the periodontitis group as shown by TRAP staining and immunohistochemistry (Fig. 3c, d). These results suggested that treatment with ABs can alleviate bone destruction by inhibiting osteoclast activity.

### miR-223-3p is the key regulator in ABs during osteoclast differentiation and bone resorption

**Fig. 4:**
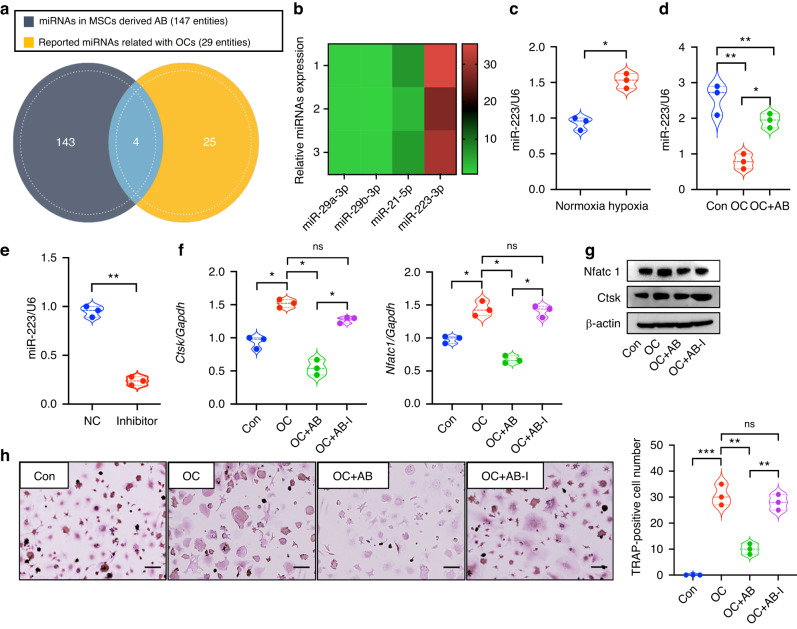
miR-223-3p is the key regulator in ABs during osteoclast differentiation and bone resorption. **a** 147 miRNAs were detected in ABs, and after comparing with the 29 miRNAs reported to be related with osteoclasts, 4 miRNAs (miR-29a-3p, miR-29b-3p, miR-21-5p and miR-223-3p) were screened. **b** miR-223-3p showed the highest expression level among the 4 screened miRNAs in ABs. **c** The expression of miR-223-3p is also upregulated in MSCs under hypoxia. **d** miR-223-3p expression is induced after the co-culture of osteoclasts with ABs. **e** An inhibitor approach was used to knock-down the expression of miR-223-3p in ABs. **f**–**h** Compared with the ABs group, there was no significant difference between the osteoclast and the AB-I groups in the expression levels of Ctsk and Nfatc1, as well as the number of TRAP-positive cells. Scale bars: 20 μm. Data are reported as means ± SD (*n* = 3). *P < 0.05, **P < 0.01, ***P < 0.001

Next, we sought to determine how ABs regulate osteoclast activity and rescue the bone destruction in periodontitis. We used microRNA-Seq analysis to detect the contents of ABs. A total of 147 miRNAs were detected in the ABs and after comparing them with miRNAs reported to be related with osteoclasts, miR-29a-3p, miR-29b-3p, miR-21-5p and miR-223-3p were identified (Fig. 4a). To identify the key components in ABs, the expression levels of those screened miRNAs were assessed. The results confirmed that miR-223-3p had the highest expression level in ABs (Fig. 4b). Coincidently, the expression of miR-223-3p was also upregulated in MSCs under hypoxia (Fig. 4c). Our previous results showed that ABs are engulfed by OCs, after which miR-223-3p is transferred into those osteoclasts since miR-223-3p expression was induced after the co-culture of osteoclasts with ABs (Fig. 4d).

A miR-223-3p inhibitor was transfected into pre-OCs and confirmed the impact of miR-223-3p on osteoclastogenesis (Fig. S4). To verify whether miR-223-3p contributes to the AB-reduced inhibition of osteoclasts, we used an inhibitor approach to knock-down the expression of miR-223-3p in ABs (Fig. 4e). miR-223-3p-silenced ABs (AB-I) were co-cultured with pre-OCs but failed to attenuate osteoclast differentiation. Compared with the ABs group, there was no significant difference between the osteoclast and the AB-I groups in the expression of Ctsk and Nfatc1 (Fig. 4f, g) or in the number of TRAP-positive cells (Fig. 4h).

**Fig. 5:**
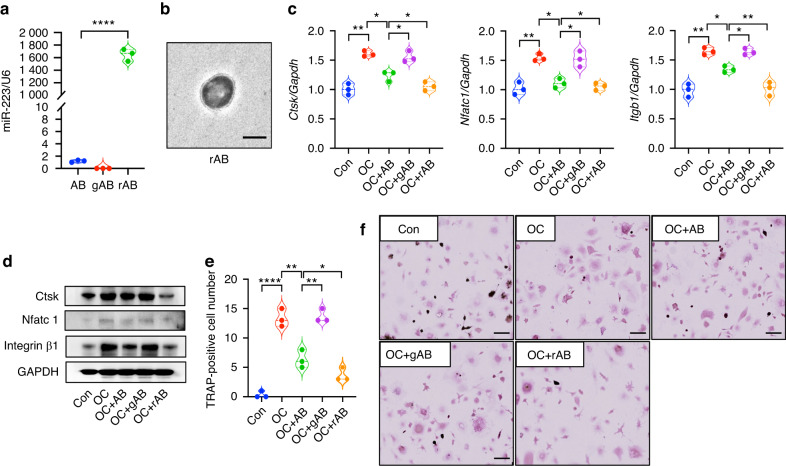
miR-223-3p enriched ABs enhanced the inhibition on osteoclasts differentiation. **a**, **b** All the contents of ABs were eliminated (gABs) and miR-223-3p enriched ABs were constructed (rABs). The expression of miR-223-3p was dramatically induced in rABs and the size of rABs was around 1 μm as assessed by TEM. **c**, **d** Compared to ABs, rABs inhibit the expression of Ctsk, Nfatc1 and Itgb1 more efficiently. **e**, **f** TRAP staining shows that rABs significantly reduce the differentiation of osteoclasts. Scale bars: 20 μm. Data are reported as means ± SD (*n* = 3). *P < 0.05, **P < 0.01, ****P < 0.000 1

To further confirm that miR-223-3p is a key factor in ABs, we constructed recombinant ABs (rABs) to eliminate the possible effects of other miRNAs in ABs. The components in ABs were excluded by centrifugation to obtain ghost ABs (gABs), which were incubated with the miR-223-3p mimic with ultrasonication after which we harvested the rABs. The expression of miR-223-3p was dramatically induced in rABs (Fig. 5a) and the size of rABs was around 1 μm as assessed by TEM (Fig. 5b). Compared to ABs, treatment with rABs inhibited the expression of Ctsk, Nfatc1 and Itgb1 more efficiently (Fig. 5c, d). TRAP staining also showed that treatment with rABs significantly reduced the differentiation of osteoclasts (Fig. 5e, f). These results confirmed the effects of miR-223-3p on osteoclasts and revealed that miR-223-3p is the key regulator in ABs during osteoclast differentiation and bone resorption.

### miR-223-3p attenuates the osteoclastogenesis process via targeting Itgb1

**Fig. 6:**
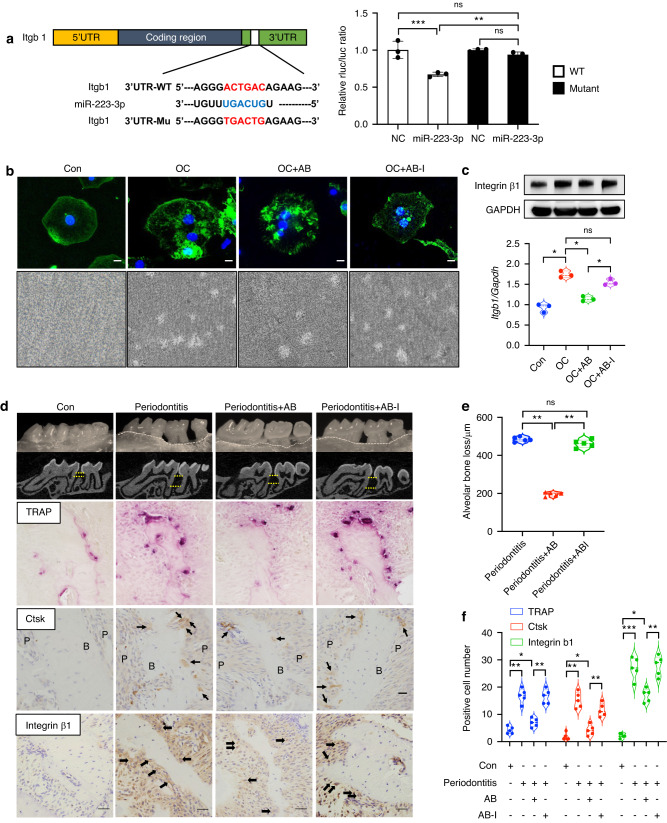
miR-223-3p attenuates the osteoclastogenesis process via the targeting of Itgb1. **a** Left, a paired target region identified between miR-223-3p and the 3′UTR of Itgb1 as predicted by Targetscan. Right, a mutation of Itgb1 was constructed according to the conserved target sites. miR-223-3p significantly decreased the luciferase activity of Itgb1-WT while Itgb1-Mu rescued that suppression. **b**, **c** FITC-phalloidin staining and Pit assays show that AB-I doesn’t affect the function of osteoclasts or the expression of integrin β1. **d**–**f** In vivo, the injection of AB-I had no effect on the rescue of bone destruction or the inability to reduce the number of osteoclasts. Upper yellow line: Cementoenamel junction; lower yellow line: alveolar crest. Black arrows: positive cells. Scale bars: 20 μm. Data are reported as means ± SD (*n* = 3). *P < 0.05, **P < 0.01, ***P < 0.001

In the present study, we characterized the interaction between miR-223-3p and Itgb1. Targetscan was used to predict miR-223-3p binding regions in the 3′UTR of Itgb1 and a paired target region was identified (Fig. 6a, left). A mutation of Itgb1 was constructed according to the conserved target sites. We found that miR-223-3p significantly decreased the luciferase activity of Itgb1-WT and that Itgb1-Mu rescued that suppression (Fig. 6a, right). FITC-phalloidin staining showed that AB-I had no impact on osteoclast maturation. Pit formation assays confirmed that AB-I didn’t affect the function of osteoclasts, since resorption pits can be formed by osteoclasts (Fig. 6b). Further, the expression of integrin β1 wasn’t suppressed by AB-I (Fig. 6c). In vivo, the injection of AB-I had no benefit in rescuing bone destruction (Fig. 6d, e) or the inability to reduce the number of osteoclasts (Fig. 6d–f).

### DC-STAMP mediates the targeted phagocytosis of ABs by osteoclasts

**Fig. 7:**
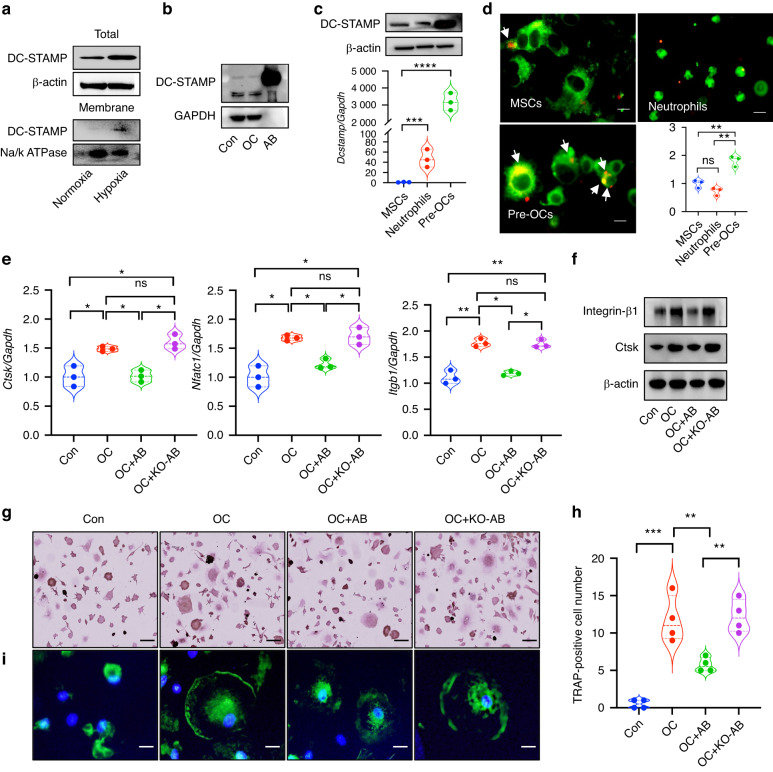
DC-STAMP mediates the targeted phagocytosis of ABs by osteoclasts. **a** The total and membrane protein expression level of DC-STAMP in MSCs is significantly induced under hypoxia. **b** Pre-OCs and ABs both express high levels of DC-STAMP. **c** The expression of DC-STAMP was the highest in pre-OCs compared with MSCs and neutrophils. **d** ABs were co-cultured with pre-OCs, stem cells and neutrophils; only a few ABs were engulfed by stem cells or neutrophils, but many ABs were phagocytosed by pre-OCs. **e**, **f** KO-ABs didn’t significantly inhibit Ctsk, Nfatc1 or Itgb1 compared with the ABs group. **g**–**i** The number of TRAP-positive cells and the sealing zone showed no difference between the KO-ABs and the osteoclast groups. Scale bars: 20 μm. Data are reported as means ± SD (*n* = 3). *P < 0.05, **P < 0.01, ***P < 0.001, ****P < 0.000 1

Since the treatment with ABs was able to ameliorate alveolar bone resorption, we asked what factor(s) mediated the targeted phagocytosis of ABs by pre-OCs. DC-STAMP is expressed on the cell membrane of osteoclasts and mediates the fusion of cell membranes, as does ATP6v0d2 and CD9. We found that the expression of DC-STAMP in MSCs was significantly upregulated by hypoxia (Figs. 7a, S5A), however, ATP6v0d2 and CD9 were suppressed under hypoxic conditions (Fig. S5A). Further, we detected the expression level of DC-STAMP in pre-OCs and in ABs and confirmed that they both expressed high levels of DC-STAMP (Fig. 7b), thus, DC-STAMP was the predicted mediator in the membrane fusion of ABs and osteoclasts. In addition to osteoclasts, stem cells and neutrophils also have the ability to phagocytose ABs. We compared the DC-STAMP expression level and affinity for ABs among those 3 kinds of cells. The expression of DC-STAMP was the highest in pre-OCs compared with stem cells and neutrophils (Fig. 7c). In addition, we co-cultured the same amounts of ABs with those 3 kinds of cells. Only a few ABs were engulfed by stem cells or neutrophils but many ABs were phagocytosed by pre-OCs (Fig. 7d). These results indicated that DC-STAMP mediates the targeted phagocytosis of ABs by osteoclasts.

To confirm whether DC-STAMP is the key mediator in the process of targeted phagocytosis, we used DC-STAMP silencing technology (Fig. S5B–F) and DC-STAMPKO mice. MSCs were isolated from DC-STAMPKO mice and ABs were harvested (KO-ABs) under hypoxia. Consistent with our previous results, KO-ABs were not engulfed by pre-OCs (Movie 1-2), and KO-ABs didn’t show any significant inhibition of Ctsk, Nfatc1 and Itgb1 compared with the ABs group (Fig. 7e, f). The number of TRAP-positive cells and the sealing zone showed no difference between the KO-ABs and osteoclast groups (Fig. 7g–i). Collectively, these findings imply that DC-STAMP is necessary for the targeted phagocytosis of ABs by osteoclasts.

### A DC-STAMP deficiency attenuates the inhibitory effect of ABs on bone destruction

**Fig. 8:**
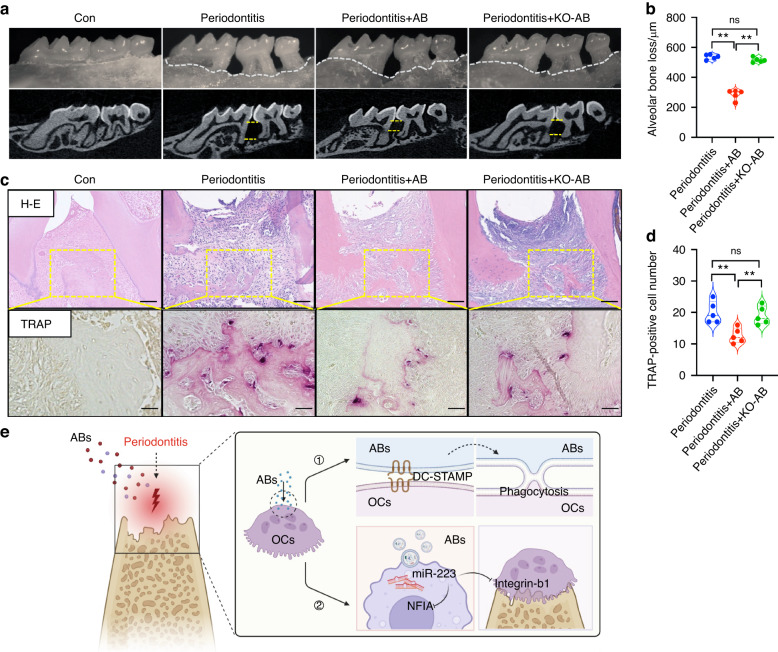
A deficiency of DC-STAMP attenuates the inhibitory effect of ABs on bone destruction. **a**, **b** The local injection of ABs reduces alveolar bone resorption, however, treatment with KO-ABs had no impact on rescuing the alveolar bone resorption compared with the periodontitis group. Upper yellow line: Cementoenamel junction; lower yellow line: alveolar crest. **c**, **d** The differentiation of osteoclasts isn’t inhibited after treatment with KO-ABs compared with the ABs treated group, as showed by TRAP staining. **e** Scheme created with BioRender showing that DC-STAMP mediates the targeted phagocytosis of ABs by osteoclasts; ABs transfer miR-223-3p to osteoclasts and inhibit the expression of NFIA and Itgb1, interfering with the differentiation of osteoclasts as well as the function of osteoclastogenesis. Finally, MSC-derived ABs have the ability to alleviate bone destruction in periodontitis. Scale bars: 20 μm. Data are reported as means ± SD (*n* = 5). **P < 0.01

As shown above, we demonstrated that DC-STAMP mediates the phagocytosis of ABs by osteoclasts in vitro. Next, we established an in vivo animal periodontitis model to validate the effects of DC-STAMP. The local injection of KO-ABs had no impact on the rescue of alveolar bone resorption compared with the periodontitis group (Fig. 8a, b). Further, the differentiation of osteoclasts wasn’t inhibited after treatment with KO-ABs (Fig. 8c, d). Taken together, these results show that DC-STAMP mediates the targeted phagocytosis of ABs by osteoclasts. Further, ABs transfer miR-223-3p into osteoclasts and inhibit the expression of NFIA and Itgb1, which interferes with the differentiation of osteoclasts as well as the function of osteoclastogenesis. Finally, MSC-derived ABs possess the ability to alleviate bone destruction in periodontitis (shown schematically in Fig. 8e, created with Biorender.com).

## Discussion

MSCs are critical for periodontal tissue regeneration, but the results of this study revealed found that a hypoxic microenvironment is one of the major reasons that induces the apoptosis of MSCs in periodontitis. During the apoptosis process, the chromatin block (nuclear fragments) formed by the condensation and fragmentation of nuclei and the membrane vesicles gradually divide into different sizes of vesicles, including exosomes, microvesicles and ABs.^33,34^ Stem cell-derived exosomes increase the expression levels of osteogenic genes, regulate the polarization of macrophages and accelerate bone formation in defects of alveolar bone;^35,36^ MSC-derived microvesicles showed a therapeutic potential for the treatment of clinical cartilage injury.^37^ ABs are the major product of apoptotic cells and contain large amounts of regulatory molecules that are involved in a wide range of biological functions. ABs can be engulfed by macrophages, fibroblasts and stem cells, which is mediated by specific interactions between phagocytes and ABs.^38,39^ Studies have shown that ABs derived from different sources of cells have different regulatory roles. Liu et al. found that circulating ABs maintain the homeostasis of MSCs and ameliorate osteopenia.^34^ MSCs-derived ABs alleviated Pg-LPS induced inflammatory responses of macrophages and regulated osteoclast formation.^40^ Osteocyte-derived ABs are capable of initiating de novo osteoclastic bone resorption.^41,42^ The role of ABs in bone homeostasis needs to be further investigated.

In this study, MSC-derived ABs effectively alleviated bone loss in an animal periodontitis model and treatment with ABs elicited a significant recovery of bone destruction. miRNAs have the characteristic of multiple targets, and a single miRNA can target multiple genes in one or more pathways to perform its biological functions compared with other factors in ABs. There were 147 miRNAs identified in ABs, in which miR-21a-5p, miR-29a-3p, miR-29a-5p and miR-223-3p were identified that are related with osteoclasts.^43–45^ miR-223-3p had the highest expression level in ABs and we confirmed that miR-223-3p is the key miRNA in osteoclast regulation. These results demonstrated that ABs suppress the differentiation and function of osteoclasts by transferring miRNA-223-3p.

Studies have shown that NFIA and IKK-α are target genes of miR-223-3p. miR-223-3p inhibited the expression of IKK-α and resulted in the suppression of Nfatc1 through the classical and non-classical NF-kB pathways, thereby inhibiting osteoclast differentiation.^46–48^ Ruffled borders and clear zones are highly polarized cytoplasmic structures of osteoclasts, which are responsible for bone resorption. Integrin and actin form the sealing zone at the mature stage of osteoclast differentiation and the integrity of the sealing zone is crucial to the function of osteoclasts.^49–52^ Integrins are transmembrane matrix receptors that have αv, α2, β1 and β3 subunits. Those subunits form heterodimer receptors that are necessary for podosome-related osteoclast adhesion and absorption functions.^53–55^ Recent studies have shown that a deficiency of integrin β1 disrupts the formation of podosomes and invadopodia by osteoclasts.^56,57^ Consistently, we found that the bone resorption ability of osteoclasts was weakened by ABs. Luciferase activity assays revealed that miR-223-3p in ABs binds to the 3′UTR of Itgb1 and inhibits its expression, which interferes with the function of osteoclasts. Thus, ABs regulate the differentiation and function of osteoclasts by transferring miR-223-3p.

DC-STAMP is preferentially expressed in myeloid DCs, macrophages and OCs, however, the expression level of DC-STAMP in osteoclasts is the highest among those cells.^58–60^ DC-STAMP is expressed as a dimer on osteoclast membranes and plays an essential and vital role in the fusion of mononuclear osteoclasts, thus increasing the absorbing activity of osteoclasts.^61^ Kukita et al. found that the overexpression of DC-STAMP on L1.2 cells induced membrane interactions with osteoclast precursors.^62^ Moreover, many multinucleated cells (≥3 nuclei per cell) were observed after transfection of the pCMV6-DC-STAMP plasmid into RAW 264.7 cells within 16 h in the absence of RANKL.^63^ Those studies indicated that intercellular membrane fusion is possible only when the cells co-express DC-STAMP, and the presence of DC-STAMP may be the inducer of its ligand. From this study, we found that osteoclast precursors and ABs express high levels of DC-STAMP on their membranes and the expression of DC-STAMP was lower in other types of phagocytes (such as stem cells and neutrophils) than in pre-OCs, which led to a restricted engulfment of ABs in these cells compared with pre-OCs. To further confirm the role of DC-STAMP, we generated DC-STAMPKO mice and harvested KO-ABs from MSCs. Surprisingly, a dynamic phagocytosis test showed that KO-ABs can not be engulfed by pre-OCs (Movie 1–2). Accordingly, KO-ABs failed to regulate the differentiation and function of osteoclasts and there was no rescue of KO-ABs on the bone destruction in the animal periodontitis model. It appears that DC-STAMP mediates the phagocytosis of ABs by pre-OCs, and the existence of DC-STAMP on the membrane is necessary for the expression of its ligand. However, the putative ligand of DC-STAMP and the underlying interaction between DC-STAMP and its ligand still need further investigation.

Clinically, it is difficult to achieve periodontal tissue regeneration since the fate of MSCs is hard to predict or control. The beneficial effects of stem cell based tissue regeneration are mediated by the paracrine action of MSCs. Hence, a better understanding of the mechanisms of MSC-derived ABs has important implications for the design of novel therapeutic approaches to treat periodontitis. Further research revealed that MSC-derived ABs can effectively reduce bone loss in an animal periodontitis model. DC-STAMP mediated the engulfment of ABs by osteoclasts. ABs inhibit the differentiation and bone resorption ability of osteoclasts by transferring miR-223-3p, which suppressed the expression of NFIA and Itgb1. These results demonstrate that ABs are efficient therapeutic agents for the treatment of periodontitis, which provides a new potential cell-free therapy to enhance periodontal regeneration.

## Materials and methods

### Cell culture

MSCs were flushed from the bone marrow cavities of femurs and tibias of euthanized mice with cell culture medium (20% fetal bovine serum, 2 mmol·L^−1^ L-glutamine, 100 U·mL^−1^ penicillin and 100 mg·mL^−1^ streptomycin (all from Invitrogen, Carlsbad, USA)) and were incubated at 37 °C and 5% CO_2_ in a humidified environment. MSCs at passage 2 were used in the present study.

### Isolation and characterization of ABs

MSCs were incubated in an AnaeroPack system (Mitsubishi Gas Chemical Co., Inc., Tokyo, Japan) for 72 h to induce apoptosis under hypoxia. The supernatant was collected and centrifuged for 10 min at 300 × *g* to remove cell debris. The supernatant was subsequently filtered through 5 μm and 1 μm filters to collect extracellular vesicles between 1 and 5 μm in diameter. Next, the supernatant was centrifuged at 2 000 × *g* for 20 min to pellet the ABs. The diameters of ABs were assessed using a DelsaMax Pro (Beckman Coulter, Brea, USA). The collected ABs were stained with 1 μg FITC-Annexin V (1:100, 55567, BD Bioscience, San Jose, USA) and PE-TSP1 (1:100, sc-59886, Santa Cruz, Santa Cruz, USA). ABs were defined as positive vesicles.

### Transmission electron microscopy

ABs were fixed with 1% glutaraldehyde solution for 15 min and washed 3 times with distilled water. The fixed ABs were centrifuged at 2 000 × *g* for 20 min and the supernatant was removed. Next, the samples were placed on formvar-carbon-coated copper grids (Ted Pella, Inc., Redding, USA) for 15 min. The liquid was removed, the ABs were fixed with acetic acid dioxygen glaze for 2 min and then washed 3 times with distilled water. The grids were dried and then observed using a JEM-2100F field-emission electron microscope (JEOL Ltd. Tokyo, Japan). Images were captured using a Tecnai F20 Twin TEM (FEI, Hillsboro, USA) operated at 120 kV.

### Animal model of periodontitis

Five-week-old female C57BL/6J mice were purchased from Vital River (Beijing, China) and DC-STAMP knockout (DC-STAMPKO) mice were obtained from Cyagen Biosciences (Guangzhou, China). All mice were housed in separate pathogen-free animal facilities with a 12:12-h light:dark cycle. DC-STAMPKO mice were used to obtain DCSTAMPKO ABs (KO-ABs). Twenty C57BL/6J mice were used for the animal periodontitis model and were divided into a control group (*n* = 5), a periodontitis group (*n* = 5), an ABs injection group (*n* = 5) and a KO-ABs injection group (*n* = 5). Mice were anesthetized with 1% chloral hydrate. The neck of the second molar of each mouse was ligated with 5-0 silk thread for 2 weeks to establish the periodontitis model. The mice were sacrificed by anesthesia followed by cervical dislocation. Alveolar bone resorption was assessed by micro-computed tomography (micro-CT, Bruker; tube voltage: 80 kV, tube current: 90 μA, time: 430 ms). All animal experiments were performed after approval by the Institute’s Ethics Committee of Beijing Stomatological Hospital, Capital Medical University (KQYY-202206-007).

### Immunohistochemistry and immunofluorescence staining

Sections of alveolar bones were subjected to antigen unmasking with sodium citrate buffer (pH 6.0), after which they were incubated with 10% normal blocking serum for 30 min at 37 °C. The primary antibodies (anti-Ctsk: 1:200, ab19027, Abcam, MA, USA; anti-Integrin β1, ab179471, Abcam) were placed on the slides at 4 °C overnight. The specimens were then incubated with secondary antibodies conjugated to peroxidase for 30 min at room temperature. Images were captured using a microscope (Olympus, Tokyo, Japan), and the numbers of immunostaining positive cells were calculated using ImageJ software.

### Osteoclastogenesis detection

Pre-osteoclasts (pre-OCs) were flushed and harvested from the bone cavities of mice and were cultured with 10% fetal bovine serum, 2 mmol·L^−1^ L-glutamine (Invitrogen), 100 U·mL^−1^ penicillin and 100 mg·mL^−1^ streptomycin (Invitrogen) and 30 ng·mL^−1^ M-CSF in a-MEM (Gibco, Carlsbad, USA). Pre-OCs were seeded at 1 × 10^5^ cells per well in 12-well plates and were induced for 4 days in osteoclast differentiation medium (30 ng·mL^−1^ M-CSF, 100 ng·mL^−1^ RANKL and/or ABs). Tartrate-resistant acid phosphate (TRAP) staining (Kamiya Biomedical Company, Seattle, USA) was used to detect osteoclast differentiation. Pit assays (Corning, NY, USA) and Phalloidin staining (Abcam) were used to detect the bone resorption ability of osteoclasts. Images were captured using a microscope (Olympus) and TRAP-positive osteoclasts were counted.

### Manipulation of ABs

The ABs were subjected to Hypotonic Lysis Buffer (Chromatrap, WXM, Wrexham, UK) at 4 °C for 1 h, and were then sonicated for 5 s (VCX 130 PB, Sonics, Newtown, USA). After centrifugation at 100 g for 10 min to remove debris, the supernatant was centrifuged at 10 000 × *g* for 10 min to concentrate ghost ABs (gABs). The gABs were incubated with the miR-223-3p mimics and were sonicated for 2 min in a bath sonicator (SY25­12, Shengyuan Supersonic, Shanghai, China), then the recombinant ABs (rABs) were centrifuged at 5 000 × *g* for 10 min. The morphology of rABs was determined using TEM (JEOL Ltd., Tokyo, Japan).

### Quantitative reverse transcription-PCR (qRT-PCR)

Total RNA was extracted from MSCs using TRIzol reagent (Invitrogen) according to the manufacturer’s instructions. Two μg aliquots of RNA were synthesized using random hexamers or oligo (dT) and reverse transcriptase for real-time PCR. The primers used for qRT-PCR are listed in Table S1. The reactions were performed using a QuantiTect SYBR Green PCR kit (Qiagen, Duesseldorf, Germany) and an IcycleriQ Multi-color Real-time PCR Detection System (Bio-Rad, Hercules, USA).

### Western blot analysis

Lysis Buffer was used to extract total proteins and 20 µg protein was used for each Western blot using standard protocols. The primary antibodies used are listed in Table S2. The membranes were incubated with horseradish peroxidase-conjugated anti-mouse/rabbit IgG (1:2 000; 98164, Cell Signaling Technology, Danvers, USA) at room temperature for 1 h. An ECL Plus Western Blotting Detection System (GE Healthcare, Buckinghamshire, UK) was used to visualize and capture images of bound antibodies.

### microRNA array

For miRNA analysis of ABs, 1.5 mg of each sample was subjected to SurePrint G3 mouse miRNA 8 × 60 K arrays (Rel. 21.0, Agilent, Santa Clara, USA) and the data were analyzed using Agilent GeneSpring GX software.

### Luciferase activity

TargetScan and miRDB were used to predict binding sites between miR-223-3p and Itgb1. A mutant vector of the Itgb1 3′-UTR in the seeding sequence was constructed according to specified base-pairing rules. HEK 293T cells (1.0 × 10^4^ cells per well) were treated in 96-well plates with Lipofectamine™ 6000 (Thermo Fisher Scientific, Waltham, USA) following the manufacturer’s instructions for transient transfection. The cells were co-transfected with the non-target control or the miR-223-3p mimics. Reporter assays used a Dual-Glo Luciferase Reporter Assay system (E1910; Promega Corp., Madison, USA) at 48 h post-transfection. All experiments were performed in triplicate and means and standard deviations were calculated.

### Statistical analysis

Statistical analyses were performed using SPSS 19.0 software. Statistical significance (*P*-value < 0.05) was assessed by an independent two-tailed Student’s t-test or analysis of variance (ANOVA).

### Supplementary information

Supplementary file-clean

Movie-1

Movie-2

Figure 1

Figure 2

Figure 3

Figure 4

Figure 5

## Data Availability

The datasets used and analyzed in this study are available from the corresponding authors (drguolijia@163.com or lililiuyi@163.com) on reasonable request.
