# Supplementary material for: Mesenchymal stem cell-derived apoptotic bodies alleviate alveolar bone destruction by regulating osteoclast differentiation and function
Source: Int J Oral Sci. 2023 Dec 1;15:51. doi: 10.1038/s41368-023-00255-y (PMC10692139; doi:10.1038/s41368-023-00255-y)
Supplement: Supplementary file 1 — Supplementary file-clean [file 41368_2023_255_MOESM1_ESM.docx]

**SUPPLEMENTARY METHODS**

**Flow cytometry analysis**

BMMSCs and pre-osteoclasts were harvested and fixed with 80% methanol. The cells were incubated in PBS containing 10% normal goat serum and 0.3 M glycine to block non-specific protein binding sites. For BMMSCs identification, the cells were incubated with PE anti-CD44, FITC anti-CD146 and APC anti-CD45 antibodies (1 μg/10^6^ cells, eBioscience, San Diego, USA). For pre-osteoclasts identification, the cells were incubated with APC anti-CD14 and PE anti-RANK (eBioscience, 1 μg/10^6^ cells). Cell preparations were immediately analyzed using flow cytometry (FACSCalibur, BD Bioscience). These experiments were performed at 4 °C in the dark.

**Apoptosis detection**

The apoptosis of BMMSCs was assessed using a FITC Annexin V Apoptosis Detection Kit I (BD Biosciences) and was performed according to the manufacturer’s instructions. Briefly, BMMSCs were collected and stained with Annexin V and/or PI for 15 min at room temperature. Binding Buffer was then added to stop the reaction and staining was analyzed by flow cytometry within 1 h.

**Tunel staining**

1 × 10^5^ BMMSCs were seeded in 6-well plates. The cells were washed with PBS and then fixed with 4% paraformaldehyde for 30 min, followed by the addition of 0.3% Triton X-100 for 5 min after which Tunel staining solution was added for 1 h at 37 ℃. The images were captured using a microscope (Olympus).

**siRNA transfection**

1 × 10^5^ pre-osteoclasts were seeded in 6-well plates. A scrambled siRNA (negative control, sc-36869, Santa Cruz Biotechnology) or a DC-STAMP siRNA (sc-142887, Santa Cruz Biotechnology) were transfected into pre-osteoclasts using RNAiMAX (Thermo Fisher Scientific). The cells were incubated under hypoxia for 72 h and the supernatant was collected for AB-siRNA isolation by a sequential centrifugation followed by a sequential filtering. The cells were harvested after transfection and analyzed by RT‐PCR and western blot.

**miRNA inhibitor transfection**

1 × 10^5^ pre-osteoclasts were seeded in 6-well plates and Corning Osteo Assay Surface wells. Twenty nM negative control or miR-223-3p inhibitor (miR20000665-1-5, RiboBio Co., Ltd., Guangzhou, China) were transfected to pre-osteoclasts using the Lipofectamine™ RNAiMAX Transfection Reagent (Thermo Fisher), followed by osteoclast induction for 5 days.

**SUPPLEMENTARY RESULTS**

**The identification of pre-OCs and BMMSCs**

Pre-osteoclasts (pre-OCs) and BMMSCs were identified by Flow cytometry. Pre-osteoclasts expressed CD14 and RANK (Fig. S1A), BMMSCs were positively expressed CD146 and CD44, negatively expressed CD45 (Fig. S1B).

**Hypoxia induced BMMSCs apoptosis**

Flow cytometry and Tunel staining results showed that the hypoxic environment significantly induced the apoptosis of BMMSCs at 72 h and there was no necrosis under hypoxia conditions (Fig. S2A-D).

**ABs iddentification**

The isolated ABs ranged from 1 to 5 μm in diameter assessed by particle size detection and by TEM, and were positive for Annexin V and TSP1 antibody staining as well (Fig. S3A,B).

**The downregulation of miR-223-3p induced osteoclast differentiation**

A miR-223-3p inhibitor was transfected into pre-OCs to investigate the impact of miR-223-3p on osteoclastogenesis (Fig. S4A). The downregulation of miR-223-3p elevated the number of TRAP-positive cells (Fig. S4B,C) and induced the expression of CTSK and Nfatc1 (Fig. S4D-E). However, the inhibition of miR-223-3p didn’t show a significant effect on the bone resorption ability of osteoclasts, as shown by FITC-phalloidin staining and the Pit assay (Fig. S4F). The expression of integrin β1 did not show a significant difference between the osteoclast groups (Fig. S4G). Thus, our results confirmed that miR-223-3p had a negative regulatory role during osteoclast differentiation.

**DC-STAMP mediated the phagocytosis of ABs**

The expression of DC-STAMP in MSCs is significantly upregulated in hypoxia while ATP6v0d2 and CD9 are suppressed under hypoxic conditions (Fig. S5A). To further verify the mediator role of DC-STAMP in the phagocytosis of ABs, DC-STAMP silencing technology was used. We transfected a DC-STAMP siRNA into BMMSCs and then isolated ABs (ABs-siRNA) (Fig. S5B). The down-regulation of DC-STAMP in BMMSCs led to the knock-down of DC-STAMP in ABs. The AB-siRNA was difficult to be phagocytosed by pre-OCs compared to ABs (Fig. S5C), followed by a reduced ability to regulate osteoclast differentiation (Fig. S5D-F). These results indicated that DC-STAMP mediates the targeted phagocytosis of ABs by osteoclasts.

**SUPPLEMENTARY FIGURE LEGENDS**

**Fig. S1** The identification of pre-OCs and BMMSCs. **(A)** Flow cytometry showed that Pre-osteoclast expressed CD14 and RANK. **(B)** BMMSCs were positively expressed CD146 and CD44, negatively expressed CD45.

**Fig. S2** Hypoxia induces cell apoptosis. **(A-D)** *In vitro* flow cytometry and Tunel staining analysis confirmed that a hypoxic environment significantly induced the apoptosis of BMMSCs at 72 h but there was no necrosis under hypoxia conditions. Scale bars: 20 μm. Data are reported as means ± SD (n = 3). * *p* < 0.05, ****p* < 0.001, *****p* < 0.0001.

**Fig. S3** ABs identification. **(A)** The isolated ABs ranged from 1 to 5 μm in diameter assessed by particle size detection and by TEM. **(B)** ABs were positive for Annexin V and TSP1 antibody staining. Scale bars: 20 μm.

**Fig. S4** miR-223-3p inhibits the differentiation of osteoclasts. **(A-E)** The downregulation of miR-223-3p elevated the number of TRAP-positive cells and induced the expression of CTSK and Nfatc1. **(F)** The inhibition of miR-223-3p didn’t have a significant effect on the bone resorption ability of osteoclasts, as shown by FITC-phalloidin staining and Pit formation assay. **(G)** The expression of integrin β1 wasn’t significantly different between the osteoclast groups. Scale bars: 20 μm. Data are reported as means ± SD (n = 3). * *p* < 0.05, ***p* < 0.01, ****p* < 0.001.

**Fig. S5** DC-STAMP mediates the phagocytosis of ABs by osteoclasts. **(A)** The expression of DC-STAMP in MSCs is significantly upregulated in hypoxia while ATP6v0d2 and CD9 are suppressed under hypoxic conditions. **(B,C)** The down-regulation of DC-STAMP in BMMSCs led to the knock-down of DC-STAMP in ABs. The AB-siRNA was difficult to be phagocytosed by pre-OCs compared to ABs. **(D-F)** AB-siRNA showed a reduced ability to inhibit the expression of CTSK, Nfatc1 and Itgb1, as well as TRAP-positive cells. Scale bars: 20 μm. Data are reported as means ± SD (n = 3). * *p* < 0.05, ***p* < 0.01, ****p* < 0.001.

**SUPPLEMENTARY MOVIE LEGENDS**

**MOVIE 1** Dynamic observation of the ABs engulfment by pre-OCs. BMMSCs derived ABs can be targeted recognized and engulfed by pre-OCs. ABs were stained with PKH26 (Red). Magnification: 10x.

**MOVIE 2** Dynamic observation of the KO-ABs engulfment by pre-OCs. BMMSCs from DC-STAMPKO mice derived KO-ABs failed to be engulfed by pre-OCs. KO-ABs were stained with PKH67 (Green). Magnification: 10x.

**TABLE S1** Primers for Quantitative Reverse Transcription-PCR.

| **Gene symbol** | **Sequence (5’-3’)** |
| --- | --- |
| Ctsk | F: gaggcggctatatgaccact |
|  | R: tcagagtcaatgcctccgtt |
| Nfatc1 | F: gaagactgtctccaccacca |
|  | R: ttctggaagcaacgggatct |
| Trap | F: agtgctgtgatttgtgccag |
|  | R: tgttccactgggctgagaat |
| Itgb1 | F: ggtcagcaacgcatatctgg |
|  | R: cattcctccagccaatcagc |
| Dcstamp | F: ccgagctgcattcctaaacc |
|  | R: gcttcgcatgcaggtattca |
| ATP6v0d2 | F: acctagtgcagtgtgagacc |
|  | R: agccaggaagttgccatagt |
| CD9 | F: acttcatcttctggctcgct |
|  | R: tctgagagtcgaatcggagc |
| Gapdh | F: atggtgaaggtcggtgtgaa |
|  | R: cattctcggccttgactgtg |

F: Forward; R: Reverse

**TABLE S2** Antibodies for Western Blot analysis.

| **Antibody** | **Dilution** | **Company** | **Catalog number** |
| --- | --- | --- | --- |
| HIF1-1a | 1:1000 | Proteintech | 20960-1-AP |
| Bax | 1:500 | Abcam | ab32503 |
| Cleaved casp3 | 1:1000 | Cell Signaling Technology | 9964S |
| MLKL | 1:1000 | Proteintech | 66675-1-LG |
| Nfatc1 | 1:500 | Abcam | ab25916 |
| CTSK | 1:500 | Abcam | ab19027 |
| Integrin β1 | 1:500 | Abcam | ab179471 |
| DC-STAMP | 1:500 | Novus | NBP1-79329 |
| Na/K ATPase | 1:1000 | Cell Signaling Technology | 3010S |
| β-actin | 1:1000 | Cell Signaling Technology | 3700 |
| GAPDH | 1:1000 | Cell Signaling Technology | 5174 |
