# Supplementary figures and images for: Mesenchymal stem cell-derived apoptotic bodies alleviate alveolar bone destruction by regulating osteoclast differentiation and function
Source: Int J Oral Sci. 2023 Dec 1;15:51. doi: 10.1038/s41368-023-00255-y (PMC10692139; doi:10.1038/s41368-023-00255-y)

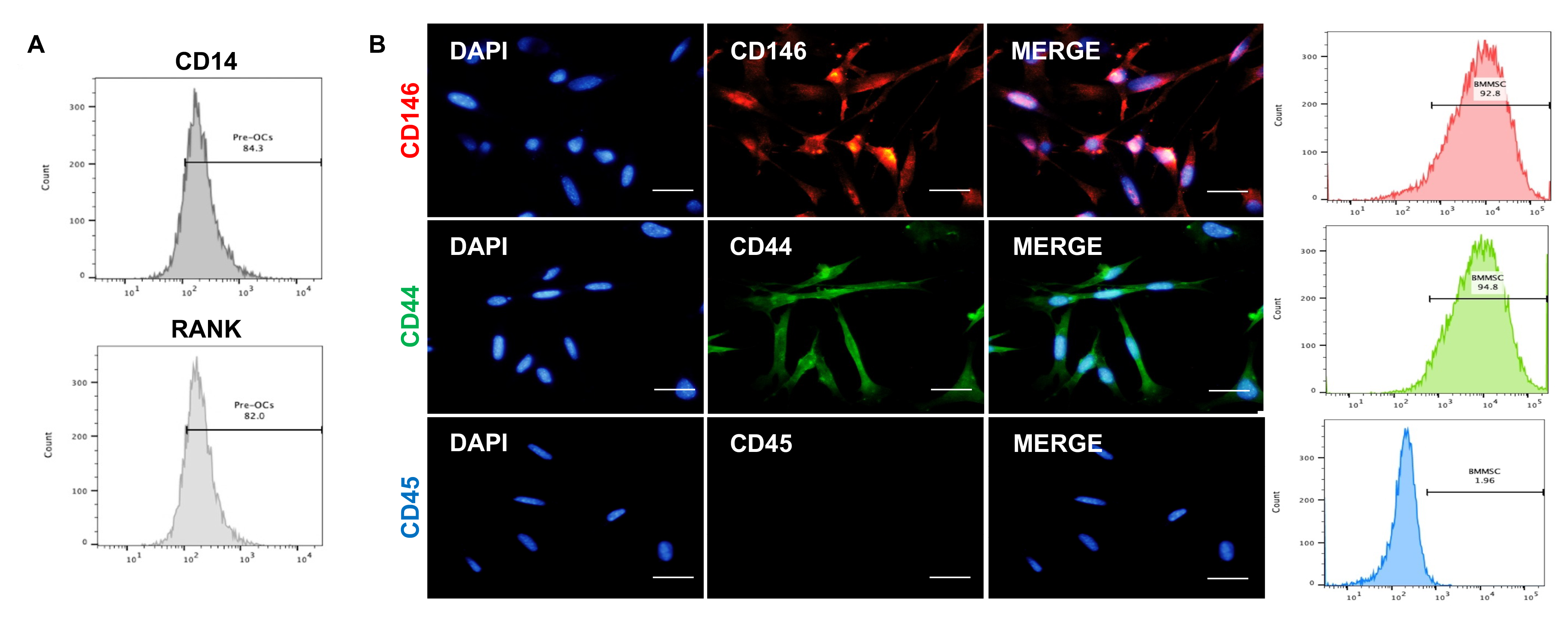

Supplement: Supplementary file 4 — Figure 1 [file 41368_2023_255_MOESM4_ESM.jpg]

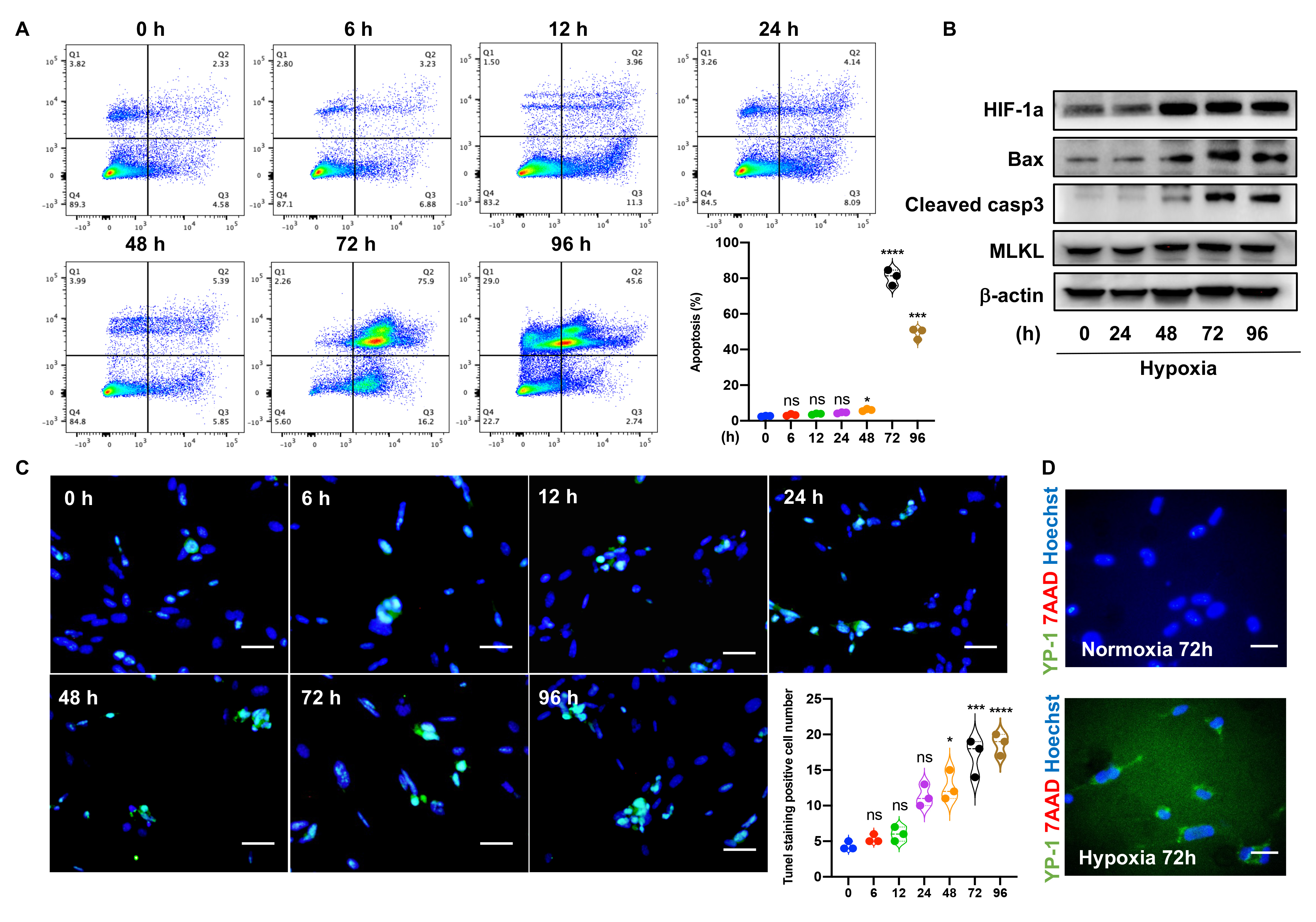

Supplement: Supplementary file 5 — Figure 2 [file 41368_2023_255_MOESM5_ESM.jpg]

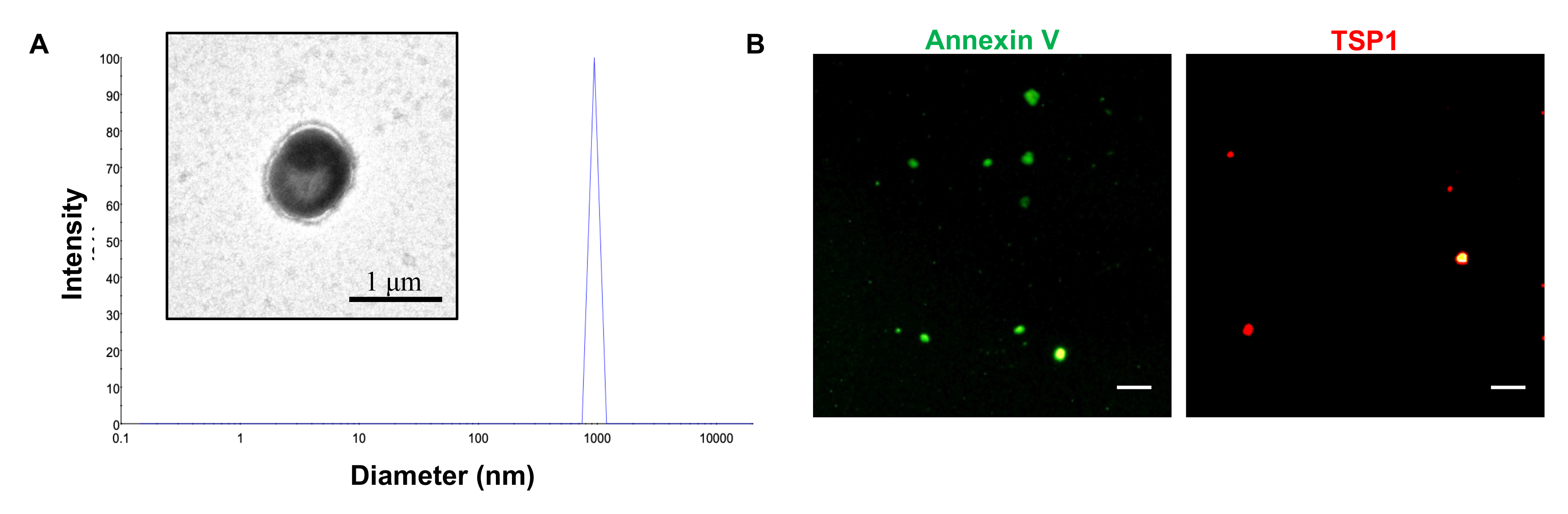

Supplement: Supplementary file 6 — Figure 3 [file 41368_2023_255_MOESM6_ESM.jpg]

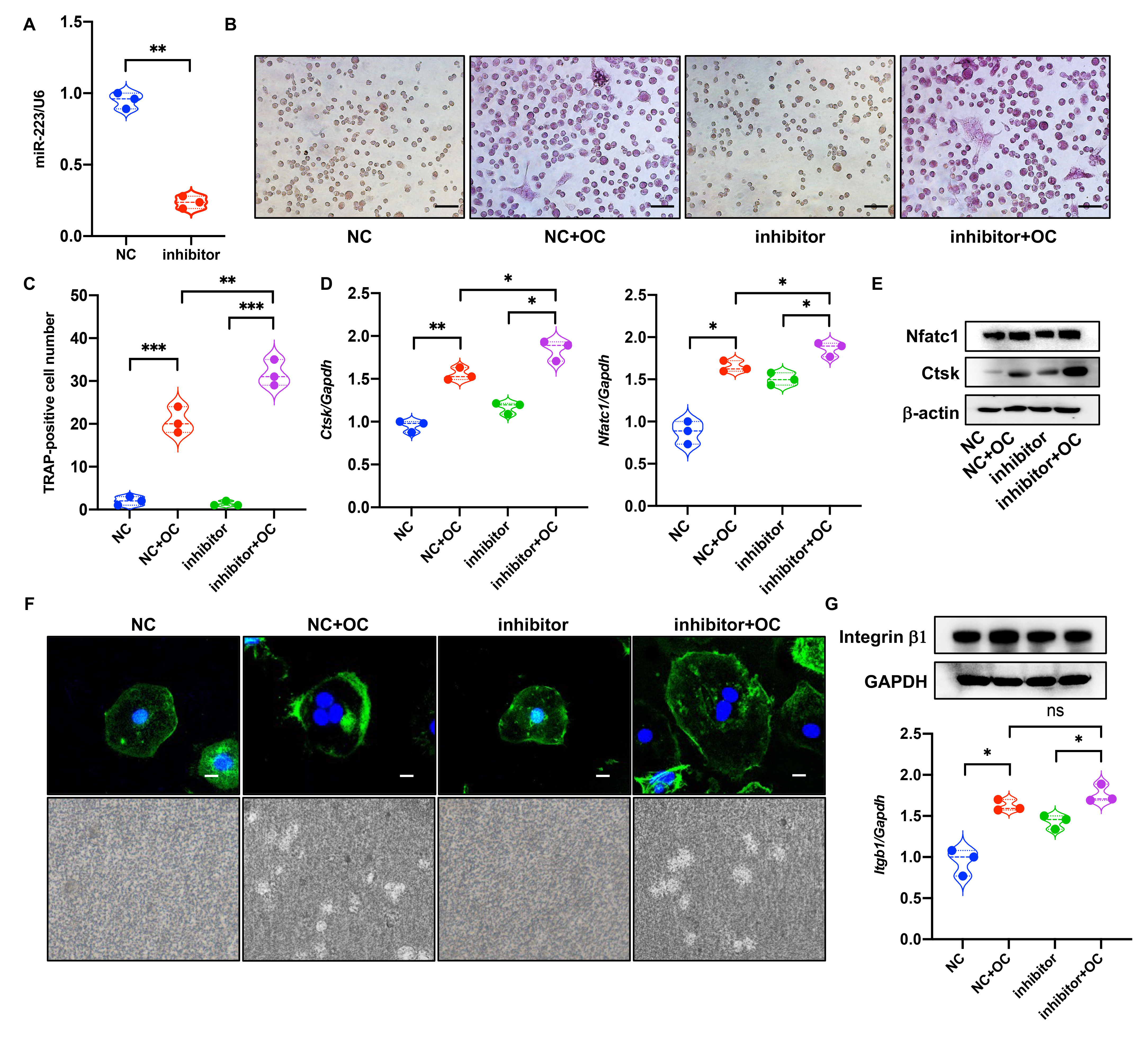

Supplement: Supplementary file 7 — Figure 4 [file 41368_2023_255_MOESM7_ESM.jpg]

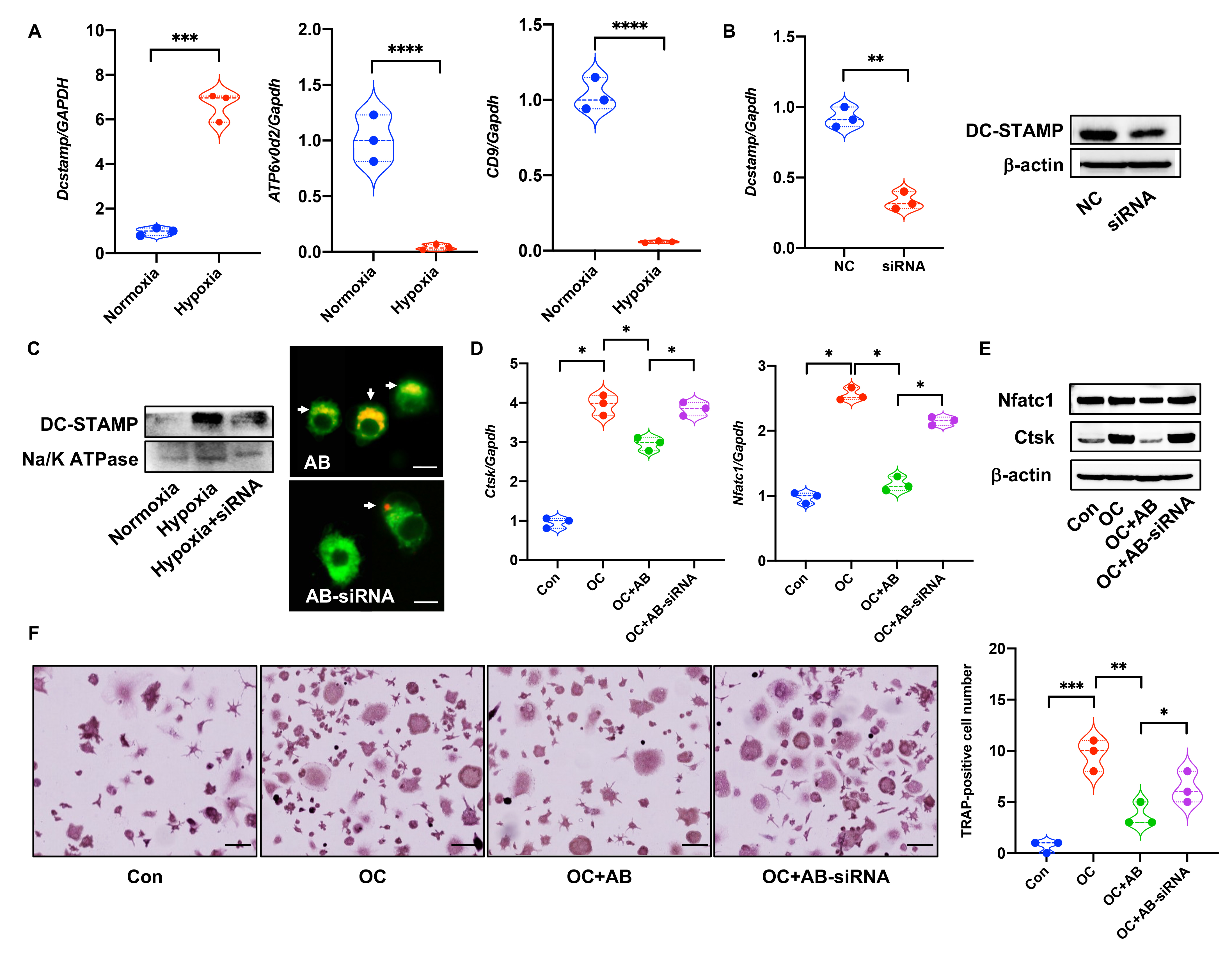

Supplement: Supplementary file 8 — Figure 5 [file 41368_2023_255_MOESM8_ESM.jpg]
